# Novel highly divergent reassortant bat rotaviruses in Cameroon, without evidence of zoonosis

**DOI:** 10.1038/srep34209

**Published:** 2016-09-26

**Authors:** Claude Kwe Yinda, Mark Zeller, Nádia Conceição-Neto, Piet Maes, Ward Deboutte, Leen Beller, Elisabeth Heylen, Stephen Mbigha Ghogomu, Marc Van Ranst, Jelle Matthijnssens

**Affiliations:** 1 KU Leuven - University of Leuven, Department of Microbiology and Immunology, Rega Institute for Medical Research, Laboratory of Viral Metagenomics, Leuven, Belgium; 2 KU Leuven - University of Leuven, Department of Microbiology and Immunology, Rega Institute for Medical Research, Laboratory for Clinical and Epidemiological Virology, Leuven, Belgium; 3 University of Buea, Department of Biochemistry and Molecular Biology, Biotechnology Unit, Molecular and cell biology laboratory, Buea, Cameroon

## Abstract

Bats are an important reservoir for zoonotic viruses. To date, only three RVA strains have been reported in bats in Kenya and China. In the current study we investigated the genetic diversity of RVAs in fecal samples from 87 straw-colored fruit bats living in close contact with humans in Cameroon using viral metagenomics. Five (near) complete RVA genomes were obtained. A single RVA strain showed a partial relationship with the Kenyan bat RVA strain, whereas the other strains were completely novel. Only the VP7 and VP4 genes showed significant variability, indicating the occurrence of frequent reassortment events. Comparing these bat RVA strains with currently used human RVA screening primers indicated that most of the novel VP7 and VP4 segments would not be detected in routine epidemiological screening studies. Therefore, novel consensus screening primers were developed and used to screen samples from infants with gastroenteritis living in close proximity with the studied bat population. Although RVA infections were identified in 36% of the infants, there was no evidence of zoonosis. This study identified multiple novel bat RVA strains, but further epidemiological studies in humans will have to assess if these viruses have the potential to cause gastroenteritis in humans.

Rotaviruses (RVs) are major enteric pathogens causing severe dehydrating diarrhea mostly in juvenile humans and animals worldwide^1^. RVs belong to the family *Reoviridae* and the genus *Rotavirus* consists of eight species (A–H)^2^. Recently, a distinct canine RV species has been identified and tentatively named *Rotavirus I* (RVI), although ratification by the International Committee on Taxonomy of Viruses (ICTV) is pending^3^. Group A rotaviruses (RVAs) are the most common of all rotavirus species and infect a wide range of animals including humans^4^. The rotavirus genome consists of 11 double-stranded RNA segments encoding 6 structural viral proteins (VP1–VP4, VP6, and VP7) and 6 nonstructural proteins (NSP1–NSP6). In 2008, a uniform classification system was proposed for each of the 11 gene segments resulting in G-, P-, I-, R-, C-, M-, A-, N-, T-, E- and H-genotypes for the VP7, VP4, VP6, VP1–VP3, NSP1–NSP5 encoding gene segments, respectively^5^,^6^. This classification system enabled researchers to compare genotype constellations between different host species and infer evolutionary patterns. In recent years host specific genotype constellations have been determined for pigs, ruminants, horses and cats/dogs^7^,^8^,^9^,^10^.

A rich but, until recently, underappreciated reservoir of emergent viruses are bats. They make up to 20% of the ∼5,500 known terrestrial species of mammals^11^ and are the second most abundant mammals after rodents^12^. Several viruses pathogenic to humans are believed to have originated from bats, including Severe Acute Respiratory Syndrome (SARS), Middle East Respiratory Syndrome (MERS)-related coronaviruses, as well as *Filoviridae*, such as Marburgvirus, and Henipaviruses, such as Nipah and Hendra virus^13^,^14^,^15^. In the last decades advances in viral metagenomics including high-throughput next-generation sequencing technologies have led to the discovery of many novel viruses including enteric viruses from bats^15^,^16^. However, rotaviruses have only been reported sporadically in bats and only three RVA strains have been characterized so far. The first strain was reported in a straw-colored fruit bat (*Eidolon helvum)* in Kenya. This partially sequenced strain was named RVA/Bat-wt/KEN/KE4852/2007/G25P[6], and possesses the following genotype constellation: G25-P[6]-I15-Rx-C8-Mx-Ax-N8-T11-E2-H10^17^. Two other bat RVAs were found in China in a lesser horseshoe bat (*Rhinolophus hipposideros*) and a stoliczka’s trident bat (*Aselliscus stoliczkanus*) named RVA/Bat-tc/CHN/MSLH14/2012/G3P[3] and RVA/Bat-tc/CHN/MYAS33/2013/G3P[10], respectively^18^,^19^. Phylogenetic analysis showed that strains MSLH14 and MYAS33, although sampling sites were more than 400 km apart, shared the same genotype constellation (G3-P[x]-I8-R3-C3-M3-A9-N3-T3-E3-H6) except for the P genotype which was P[3] for MSLH14 and P[10] for MYAS33.

To further study the genomics of RVA in bats and their zoonotic potential in humans, we screened stool samples of straw-colored fruit bats (*Eidolon helvum*) living in close proximity with humans in the South West Region of Cameroon (Fig. 1), as well as samples from infants with gastroenteritis. Our choice of this region is due to the fact that bats are considered a delicacy and the species sampled are the most commonly eaten bat species in these localities.

## Results

### Sample characterization

A total of 24 pools of 3–5 bat fecal samples were constituted, enriched for viral particles and sequenced. Illumina sequencing yielded between 1.1 and 7.8 million reads per pool, and DIAMOND classification^20^ of the obtained contigs indicated that five pools contained a significant amount of RVA sequence reads. The percentage reads mapping to RVA in each pool ranged from 0.1–2.4% (Table 1). Partial segments were completed by regular PCR and Sanger sequencing, to obtain at least the entire ORF for each of the obtained variants. Obtained sequences were used for phylogenetic comparison with a selection of representative members of each genotype. The RVA strains discovered in this study were named RVA/Bat-wt/CMR/BatLi08/2014/G31P[42], RVA/Bat-wt/CMR/BatLi09/2014/G30P[42], RVA/Bat-wt/CMR/BatLi10/2014/G30P[42], RVA/bat-wt/CMR/BatLy03/2014/G25P[43] and RVA/Bat-wt/CMR/BatLy17/2014/G30P[47] hereafter referred to as BatLi08, BatLi09, BatLi10, BatLy03 and BatLy17, respectively. All the obtained sequences were highly divergent from established genotypes and were therefore submitted to the Rotavirus Classification Working group (RCWG) for novel genotype assignments (see below) and to GenBank (accession numbers: KX268743–KX268797).

### Phylogenetic analysis

The VP7 gene of BatLy03 was 96% identical (on the nucleotide (nt) level, supplementary data S1) to the Kenyan bat RVA strain KE4852 counterpart, which had been previously classified as a G25 genotype (Fig. 2). BatLi10 and BatLi09 were 100% identical and also clustered closely with strain BatLy17 (92% similar). This cluster was only distantly related to all other known VP7 RVA sequences as well as to strain BatLi08, which also formed a unique long branch in the phylogenetic tree. Both clusters only show similarities below 74% with established genotypes (Fig. 2). The VP7 of these 4 strains (BatLi10, BatLi09, BatLy17 and BatLi08) did not belong to any of the established RVA G-genotypes, according to the established criteria^6^, and were assigned genotypes G30 (BatLi09, BatLi10 and BatLy17) and G31 (BatLi08) by the RCWG. For VP4, VP1 and VP3, all five Cameroonian bat RVAs strains were distantly related to other known RVA strains, including the Kenyan and Chinese RVA strains and were therefore assigned to novel genotypes according to the RCWG classification criteria (Fig. 3). The VP4 gene of strains BatLi08, BatLi09 and BatLi10 (representatives of the novel genotype P[42]) were almost 99–100% identical to each other and only 56–75% identical to any other P-genotype. That of strains BatLy03 and BatLy17 had 29% nt dissimilarity to each other and their nt identity ranged from 60–76% with other P-genotypes and therefore were assigned the genotypes P[43] and P[47], respectively. The VP1 and VP3 genes of BatLi08, BatLi09, BatLi10 and BatLy17 were nearly identical (nt identity range 99–100%) and clustered together but distinct from other established R and M-genotypes, thereby representing the new genotypes R15 and M14, respectively (Fig. 3). The VP1 and VP3 genes of BatLy03 were only distantly related to the other four Cameroonian bat RVAs (68–77% nt identity) and are the sole member of the newly assigned genotypes R16 and M15, respectively (Fig. 3). The VP6, VP2, NSP2, NSP3 and NSP5 gene segments of 4 of our strains (BatLi08, BatLi09, BatLi10 and BatLy17) were distantly related to their counterparts of other mammalian and avian RVAs (Fig. 4). For all the 4 strains, these gene segments clustered together and were 99–100% identical to each other and consequently they constitute new genotypes for the different gene segments (I22, C15 N15, T17 and H17, respectively). The VP6, VP2, NSP2, NSP3 and NSP5 gene segments of BatLy03 phylogenetically clustered together with the Kenyan bat RVA strain KE4852 in the previously established I15, C8, N8, T11 and H10 genotypes, respectively (Fig. 4). For NSP1, the Cameroonian bat strains BatLi08, BatLi09, BatLi10 and BatLy17 clustered closely together (98–100% nucleotide sequence identity) in the novel genotype A25, and showed only 67% nucleotide similarity to strain BatLy03 (A26). These 5 new NSP1 gene segments were only 42–45% identical to that of the most closely related established NSP1 genotype A13 and A14 (from cow) (Fig. 5). The NSP4 gene segments of all the 5 RVAs discovered in this study were quite divergent to those of other known bat rotaviruses (at most 69% nucleotide sequence identity) and other RVAs (approximately 45–68% nucleotide similarity) forming two distinct clusters. The NSP4 gene segments of strains BatLi08, BatLi09, BatLi10 and BatLy03 (genotype E22) were 100% identical but all were 37–38% divergent from that of BatLy03 (E23, Fig. 5).

### Bat rotaviruses in humans?

Several different primer pairs are currently being used to detect human RVA VP7 and VP4 gene segments, to determine the G- and P-genotypes using sequencing or multiplex PCR assays^21^,^22^,^23^,^24^. In order to find out if the currently used human RVA screening primers would detect the bat RVA strain from this study in case of zoonosis, we compared these primers with their corresponding sequences in the respective gene segments (Table 2 and Supplementary data S2). Overall, the similarity percentages for the VP7 forward and reverse primer between the bat RVA sequences and the human primers were 57.1–100% and 63.2–95%, respectively. For VP4, the percentage similarity ranged from 63.6–94.4% and 76.2–85.7% for the forward and reverse primers, respectively. VP7 forward primers Beg9, sBeg9 and 9Con1-L showed a (near) perfect match with BatLy03-G25, whereas strain BatLi08-G31 and BatLy17-G30 (first 6 nt are missing for this strain), showed up to 10 and 4 nucleotide mismatches at the 3′end of the primers, respectively. VP7 forward primer 9con1-L showed a perfect match with all the genotypes (G25, G30 and G31). Considering the VP7 reverse primers EndA, VP7-Rdeg, End9 and RVG9, BatLy03-G25 did not show a perfect match as there were 4, 1, 4 and 2 mutations, respectively. The mismatches with EndA, VP7-Rdeg and RVG9 were near the middle or at the 5′end of the primer, whereas 2 of those of End9 were close to the 3′end. Comparing the same VP7 reverse primers with strain BatLi08-G31 and BatLi09-G30 also showed mismatches. For EndA and VP7-Rdeg maximum 2 mismatches are located in the middle or near the 5′-end, whereas for End9 and RVG, there were multiple mismatches of which 2 and 7 mismatches, respectively were right at the 3′-end. For VP4 forward primer VP4-1-17F, BatLy03-P[43], BatLi08-P[42], BatLy17-P[47] and BatLi09-P[42] showed 2, 2, 1 and 2, mismatches, respectively, with at least a mutation at the first position from the 3′end for all of them. For con3, there were 6, 7, 6 and 8 mismatches with BatLy03-P[43], BatLi08-P[42), BatLi09-P[42] and BatLy17-P[47], respectively. Considering the VP4 reverse primer con2, strains BatLy03-P[43], BatLi08-P[42], BatLi09-P[42], BatLy17-P[47] and BatLi10-P[42] showed 3–5 mismatches including one at the second position from the 3′-end.

Additionally, to determine if any of these bat RVAs could cross species and infect humans, we designed primers (RVA-VP6_40F and RVA-VP6_1063R) from an alignment of both human and bat RVA VP6 segments to screen 25 diarrheic infant samples (infants living around the same region where the bat samples were collected). Thirty-six percent of human samples were positive for RVA, however, none of them was of bat RVA origin. They all possessed the typical human genotype I1 and were 99% identical to the Gambian, Senegalese, Belgian and Brazilian Wa-like G1P[8] strains BE00007 (HQ392029), MRC-DPRU3174 (KJ752288), MRC-DPRU2130-09 (KJ751561), rj1808-98 (KM027132), respectively (Supplementary data S3).

In summary, strain RVA/Bat-wt/CMR/BatLy03/2014/G25P[43] possessed the genotype constellation G25-P[43]-I15-R16-C8-M15-A26-N8-T11-E23-H10 (Table 3), which shared six genotypes with those of the Kenyan bat RVA strain KE4852. For VP4 (P[6] vs P[43]), VP1 (R-unassigned vs R16) and NSP4 (E2 vs E23), different genotypes were observed, whereas for VP3 and NSP1 no sequence data were available for KE4852 for comparison. The 4 other strains were named BatLi08, BatLi09, BatLi10 and BatLy17 and possessed the genome constellations: Gx-P[x]-I22-R15-C15-M14-A25-N15-T17-E22-H17 with G31P[42] for BatLi08, G30P[47] for BatLy17, and G30P[42] for BatLi09 and BatLi10 (Table 3). Screening human samples for these bat RVAs indicated no interspecies transmissions and primer comparison showed that not all the strains can be picked up with the currently used screening primers.

## Discussion

Bats have been proven to harbor several human pathogenic viruses including SARS, MERS-related coronaviruses, as well as filoviruses, such as Marburgvirus, or Henipaviruses, such as Nipah and Hendra virus^13^,^14^,^15^, but bat RVAs have only been sporadically reported. So far, only 3 bat RVA strains have been characterized and the first strain was reported in a straw-colored fruit bat in Kenya named RVA/Bat-wt/KEN/KE4852/2007/G25P[6]^17^; while the other two, named RVA/Bat-tc/CHN/MSLH14/2012/G3P[3] and RVA/Bat-tc/CHN/MYAS33/2013/G3P[10], were isolated from a lesser horseshoe bat, and a Stoliczka’s trident bat in China, respectively^18^,^19^.

**Figure 1:**
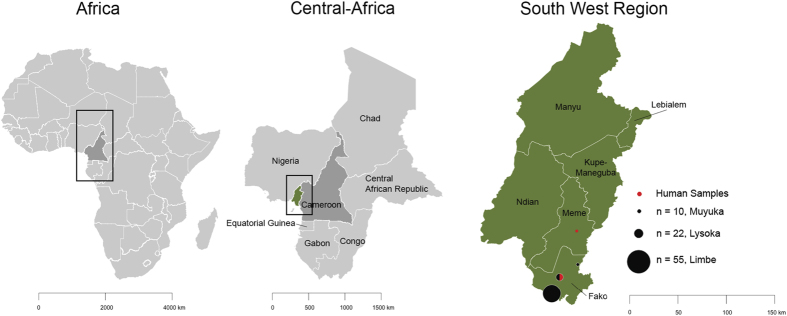
Map of study site (South West Region, Cameroon). The number of bat (black filled circles) and human (red filled circles) samples are indicated. Maps were created in R^40^ (version 3.2.3), using the raster package^41^ and the default plotting packages.

**Figure 2:**
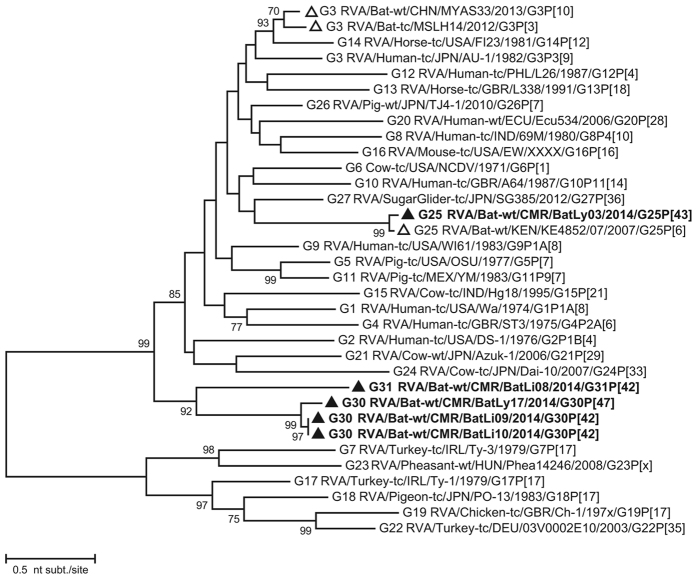
Phylogenetic trees of full-length ORF nucleotide sequences of RVA VP7. Filled triangle: Cameroonian bat RVA strains; open triangles: previously described bat RVA strains. Bootstrap values (500 replicates) above 70 are shown.

**Figure 3:**
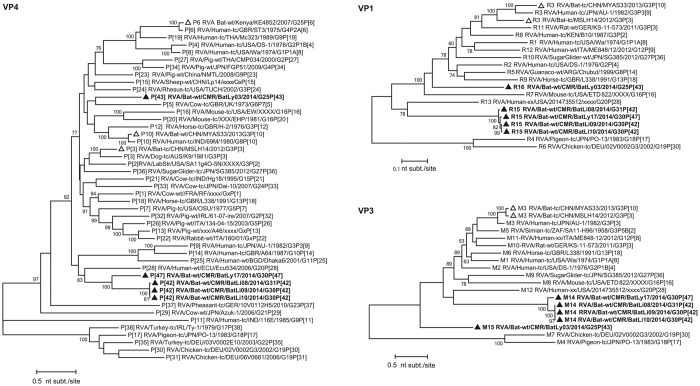
Phylogenetic trees of full-length ORF nucleotide sequences of the RVA VP4, VP1, VP3 gene segments. Filled triangle: Cameroonian strains; open triangles: previously described bat RVA strains. Bootstrap values (500 replicates) above 70 are shown.

**Figure 4:**
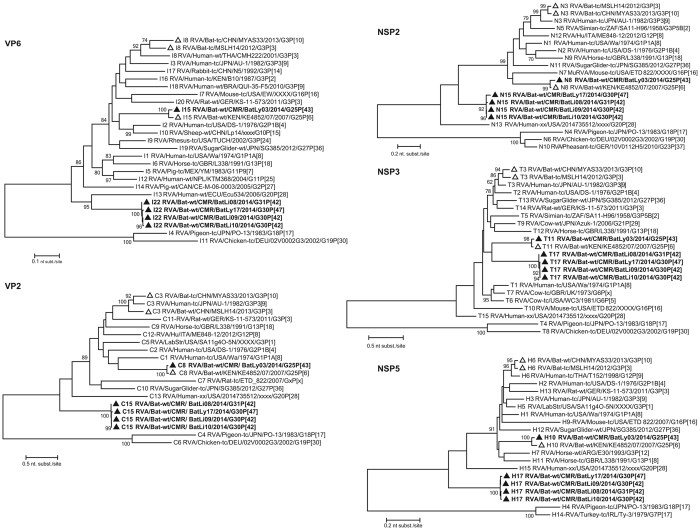
Phylogenetic trees of full-length ORF nucleotide sequences of the RVA VP6, VP2 NSP2, NSP3 and NSP5 gene segments. Filled triangle: Cameroonian strains; open triangles: previously described bat RVA strains. Bootstrap values (500 replicates) above 70 are shown.

**Figure 5:**
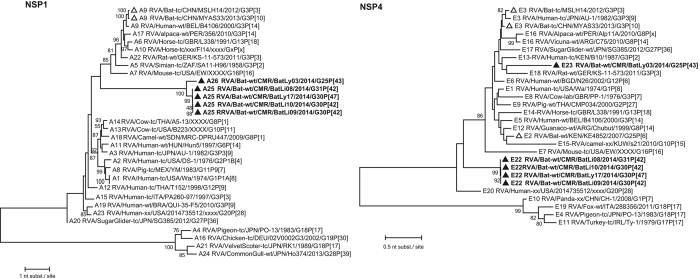
Phylogenetic trees of full-length ORF nucleotide sequences of the RVA NSP1 and NSP4 gene segments. Filled triangle: Cameroonian strains; open triangles: previously described bat RVA strains. Bootstrap values (500 replicates) above 70 are shown.

To better understand the spread and diversity of RVA in bats, we performed an RVA screening in Cameroonian bats, after trapping both male and female, young and adult bats close to human dwellings in Muyuka, Limbe and Lysoka localities of the South West region of Cameroon (Fig. 1). Using an unbiased viral metagenomics approach, we identified 5 divergent novel bat RVA strains, 4 of which were genetically similar to each other. The fifth strain was related to the Kenyan bat strain. Interestingly, all these RVAs were identified in adult (both female and male) straw-colored fruit bats (*Eidolon helvum*) which is in contrast to human and other animals whereby RVA (symptomatic) infections occur mostly in juveniles^1^. Also, diarrhea or other obvious signs of sickness were not noticed in these bats. This may suggest that bats may undergo active virus replication and shedding without obvious clinical signs^25^, which potentially could increase human exposure.

Even though there exists a considerable genetic divergence between bat RVA and human RVA, suggestions have been made about potential interspecies transmission of Chinese and Kenyan bat RVA strains. The two Chinese RVA strains are genetically quite conserved (all segments of both strains have the same genotype except for their VP4 gene). Based on genome comparisons of Chinese bat and partial human RVA strains from Thailand (CMH079 and CMH222) and India (69 M, 57 M and mcs60), Xia and colleagues speculated that Asian bat RVAs may have crossed the host species barrier to humans on a number of occasions^19^. In addition, the unusual equine strain E3198^26^ shares the same genotype constellation with either MYAS33 and/or MSLH14 in all segments except VP6. This data therefore suggests that this equine RVA strain most likely share a common ancestor with Asian bat RVAs. Furthermore, the genotype constellations of these Asian bat RVA (Table 3) are reminiscent to the Au-1-like genotype backbone of feline/canine-like RVA strains, as well as to the genotype constellation of two unusual simian RVAs (RRV and TUCH)^27^, suggesting that interspecies transmissions might have also occurred in the distant past. Moreover, an unusual Ecuadorean human RVA, Ecu534^28^ is closely related to bat sequences from Brazil recently submitted to GenBank. Similarly, possible interspecies transmission trends were also suggested by He and colleagues^18^ between bovine strain RVA/Cow-wt/IND/RUBV3/2005/G3P[3] and the bat strain MSLH14.

Given the novelty of the bat RVA strains described here, it is questionable if the currently used human RVA screening primers (for VP7 and VP4) will pick up these divergent strains in case an interspecies transmission from bats to humans would occur. Comparisons (Table 2 and Supplementary data S2) of these primers with the corresponding sequences showed that the primer combination 9Con1-L and VP7-Rdeg^21^,^22^, would most likely detect both G25 and G31 RVA strains. Also, the combination of either Beg9 or sBeg9 with End9 or RVG9^24^ might be successful in amplifying G25, but the same combinations might not be able to pick up the novel G31 and G30 genotypes in PCR screening assays. Furthermore, the primer combination Beg9/sBeg9 and EndA^29^ are likely to detect G25, but will be unsuccessful in case of G30 and G31 especially if the forward primer Beg9 is used. Considering VP4, both forward primers, VP4-1-17F^30^ and Con3^23^ in combination with the reverse primer Con2 will be sub-optimal in detecting any of the P genotypes. Generally, with the exception of strain G25, detection of most of the bat strains will be sub-optimal or not successful at all for the different available primer combinations. Therefore, zoonotic events of bat RVA strains could easily be missed with the current screening primers depending on the primer combinations, PCR conditions and/or circulating zoonotic strains.

In order to investigate the possibility of bat RVA infecting humans who are living in close contact with bats, we used novel primers (RVA-VP6_40F and RVA-VP6_1063R) designed from an alignment of both human and bat VP6 RVA segment to screen 25 infant samples from patients with gastroenteritis, living around the same region where the bat samples were collected. Interestingly, 36% of human samples were positive, however, none of these were positive for bat RVAs. All were of the typical human RVA genotype I1 and therefore there is no evidence for interspecies transmissions of bat RVA to humans. However, this result is not conclusive as only a small sample size was considered here. Sampling a larger number of subjects and from different localities around the region might result in more conclusive answers with respect to the zoonotic potential of these bat RVA strains.

The genotype constellations of the two Chinese bat RVAs showed clear indication of recent reassortment event(s) because they possessed different P genotypes (P[10] for MYAS33 and P[3] for MSLH14), and some gene segments were nearly identical whereas others were not^18^,^19^. Their genotype constellation differs markedly from the Kenyan straw-colored fruit bat strain (KE4852). Although this strain showed a unique genotype backbone, some of its segments were similar to some human and other animal RVAs^17^. Moreover, KE4852 share the same genotypes in several gene segments (VP2, VP6, VP7, NSP2, NSP3 and NSP5) with our bat RVA strain BatLy03 indicating possible reassortment events between different bat RVA strains, as well as a large geographical spread of this virus. Furthermore, BatLi08, BatLi09, BatLi10 and BatLy17 had conserved genotype constellations (in VP6, VP1–VP3, NSP1–NSP5) with 98–100% nucleotide sequence similarity except for the VP7 of BatLi08 and VP4 of BatLy17, again confirming reassortment events within bat RVAs.

The high genetic divergence and partial relatedness of most of the segments of the different bat RVA strains and the ones identified in this study indicate the frequent occurrence of reassortment events in the general bat population and those of Cameroon in particular. Also, with the current knowledge of the genetic diversity, there seems to exist several true bat RVA genotype constellations, as has been previously described for humans, and cats/dogs^10^,^31^. However, this needs to be further confirmed by identification of a larger number of RVAs from bats from different age groups and different geographical locations.

## Methods

### Ethical authorization

Ethical authorization for the use of human samples was obtained from the Cameroon National Ethics Committee, Yaoundé. All human experiments were performed in accordance with the Ministry’s National Ethics Committee guidelines. Ethical authorization for the protocol and the use of animal samples was also obtained from the Cameroon National Ethics Committee, Yaoundé. All animal experiments were performed in accordance with the Ministry’s National Ethics Committee guidelines. All experimental protocols used in this study were approved by Cameroon National Ethics Committee. Administrative authorization was obtained from the Delegation of Public Health for South West Region, Cameroon. Informed consent was obtained from human subjects or their parents or guardians.

### Bat sample collection

Bat samples were collected between December 2013 and May 2014 using a previously described method^32^. Briefly, bats were captured in 3 different regions (Lysoka, Muyuka and Limbe) of the South West Region of Cameroon (Fig. 1) using mist nets around fruit trees and around human dwellings. Captured bats were retrieved from the traps and held in paper sacks for 10–15 min, allowing enough time for the excretion of fresh fecal boluses. Sterile disposable spatulas were used to retrieve feces from the paper sacks, and placed into tubes containing 1 ml of universal transport medium (UTM, Copan Diagnostics, Brescia, Italy). Labeled samples were put on ice and then transferred to the Molecular and cell biology laboratory, Biotechnology Unit, University of Buea, Cameroon and stored at −20 °C, until they were shipped to the Laboratory of Viral Metagenomics, Leuven, Belgium where they were stored at −80 °C. Each captured bat was assessed to determine species, weight (g), forearm length (mm), sex, reproductive state, and age. All captured bats were then marked by hair clipping to facilitate identification of recaptures, and released afterwards. Trained zoologists used morphological characteristics to determine the species of the bats before they were released. No clinical signs of disease were noticed in any of these bats.

### Sample preparation for NGS

Eighty-seven fecal samples were grouped into 25 pools each containing three to five samples and treated to enrich viral particles as follows: fecal suspensions were homogenized for 1 min at 3000 rpm with a MINILYS homogenizer (Bertin Technologies, Montigny-le-Bretonneux, France) and filtered consecutively through 100 μm, 10 μm and 0.8 μm membrane filters (Merck Millipore, Massachusetts, USA) for 30 s at 1250 *g*. The filtrate was then treated with a cocktail of Benzonase (Novagen, Madison, USA) and Micrococcal Nuclease (New England Biolabs, Massachusetts, USA) at 37 °C for 2 h to digest free-floating nucleic acids. Nucleic acids were extracted using the QIAamp Viral RNA Mini Kit (Qiagen, Hilden, Germany) according to the manufacturer’s instructions but without addition of carrier RNA to the lysis buffer. First and second strand cDNA synthesis was performed and random PCR amplification for 17 cycles were performed using a Whole Transcriptome Amplification (WTA) Kit procedure (Sigma-Aldrich), with a denaturation temperature of 95 °C instead of 72 °C to allow the denaturation of dsDNA and dsRNA. WTA products were purified with MSB Spin PCRapace spin columns (Stratec, Berlin, Germany) and the libraries were prepared for Illumina sequencing using the NexteraXT Library Preparation Kit (Illumina, San Diego, USA). A cleanup after library synthesis was performed using a 1.8 ratio of Agencourt AMPure XP beads (Beckman Coulter, Inc., Nyon, Switzerland)^33^. Sequencing of the samples was performed on a HiSeq 2500 platform (Illumina) for 300 cycles (2 × 150 bp paired ends). Partial sequences were completed using RT-PCRs with specific primers (Supplementary S4). For gene segments lacking the 5′ and/or 3′ ends of the ORF the single primer amplification method (Primers in Supplementary S4) was used as described previously^34^. Sanger sequencing was done on an ABI Prism 3130 Genetic Analyzer (Applied Biosystems, Massachusetts, USA).

### Human fecal sample collection and Screening

Human fecal samples were collected from patients in Lysoka local clinic and Kumba District Hospital of the South west Region of Cameroon (Fig. 1) after informed consent was obtained from patients or their parents or guardians. The patients were either diarrheic or came into contact with bats directly (by eating, hunting or handling) or indirectly (if family member is directly exposed to bats). The samples were put in UTM containing tubes and stored the same way like the bat samples. Screening primers (Supplementary data S4) were designed from a consensus sequences of human and bat VP6 RVAs and a total of 25 samples from infants (0–3 years) who had diarrhea were screened by reverse transcriptase polymerase chain reaction (RT-PCR) using the OneStep RT-PCR kit (Qiagen). The products of positive samples were sequenced using Sanger sequencing Method.

### Genomic and phylogenetic analysis

Raw Illumina reads were trimmed for quality and adapters using Trimmomatic^35^, and were *de novo* assembled into Scaffold using SPAdes^36^. Scaffolds were classified using DIAMOND in sensitive mode^20^. Contigs assigned to RVA were used to map the trimmed reads using the Burrows-Wheeler Alignment tool (BWA)^37^. Open reading frames (ORF) were identified with ORF Finder analysis tool (http://www.ncbi.nlm.nih.gov/gorf/Orfig.cgi) and the conserved motifs in the amino acid sequences were identified with HMMER^38^. Amino acid alignments of the viral sequences and maximum likelihood phylogenetic trees were constructed in MEGA6.06^39^, using the GTR + G (VP1, VP6, NSP2 and NSP3), GTR + G + I (VP2–VP4, VP7 and NSP1), HKY (NSP4) and T92 (NSP5) substitution models (after testing for the best DNA/protein model), with 500 bootstrap replicates. Nucleotide similarities were also computed in MEGA by pairwise distance using p-distance model. Phylogenetic analyses were performed using appropriate reference strains in addition to the RVA discovered in this study.

## Additional Information

**How to cite this article**: Yinda, C. K. *et al.* Novel highly divergent reassortant bat rotaviruses in Cameroon, without evidence of zoonosis. *Sci. Rep.* **6**, 34209; doi: 10.1038/srep34209 (2016).

## Supplementary Material

Supplementary Information

## Figures and Tables

**Table 1:** Geographic location of sample collection, number of reads per pool, reads mapping to RVAs and reads mapping per gene segment.

Pool	BatLi10 (P10)	BatLyP03 (P03)	BatLi08 (P08)	BatLi09 (P09)	BatLyP17(P17)
Location	Limbe	Lysoka	Limbe	Limbe	Lysoka
No of reads/pool	7,819,081	920,217	2,140,494	1,106,398	4,446,078
No of reads mapping to RVAs	86,063	1,881	50,332	16,632	3,485
Percentage RVA reads	1.1	0.2	2.35	1.5	0.1
No of reads mapping to VP7	1,387	145	1,775	702	59
No of reads mapping to VP4	15,513	217	8,532	2,971	619
No of reads mapping to VP6	7,520	353	4,120	3,214	465
No of reads mapping to VP1	11,667	265	8,189	1,460	685
No of reads mapping to VP2	12,727	251	8,934	2,046	353
No of reads mapping to VP3	11,552	112	7,062	1,208	383
No of reads mapping to NSP1	16,200	150	5,552	2,386	554
No of reads mapping to NSP2	2,703	68	1,924	675	146
No of reads mapping to NSP3	4,855	159	2,352	1,224	100
No of reads mapping to NSP4	957	103	841	390	64
No of reads mapping to NSP5	672	18	341	356	22

**Table 2:**
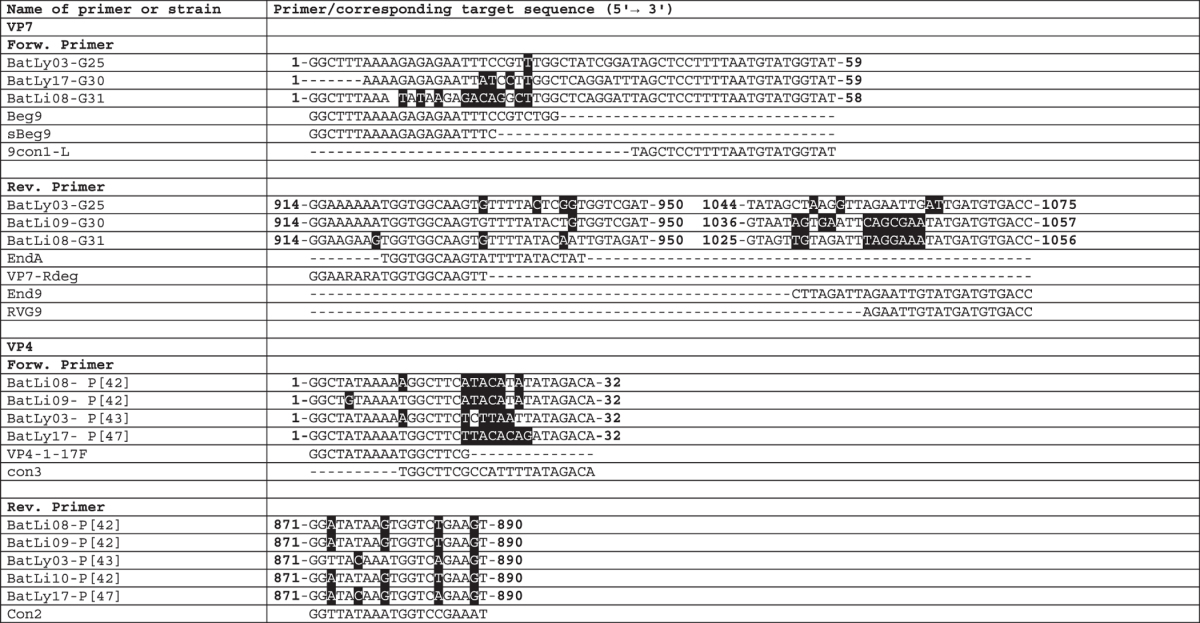
Nucleotide comparison between the sequence of human RVA screening primers for VP7 (Beg9, sBeg9, 9Con1-L, EndA, VP7-Rdeg, End9 and RVG9) and VP4 (VP4-1-17F, Con3 and Con2) with their corresponding region of the new bat RVA genotype.

Black shaded nucleotides indicate dissimilar nucleotides between a strain segment sequence and primer and bold numbers at the beginning and end of sequence indicate nucleotide positions for different strains. For clarity the reverse complement sequence of the reverse primers is used for comparison with bat RVA sequences.

**Table 3:**
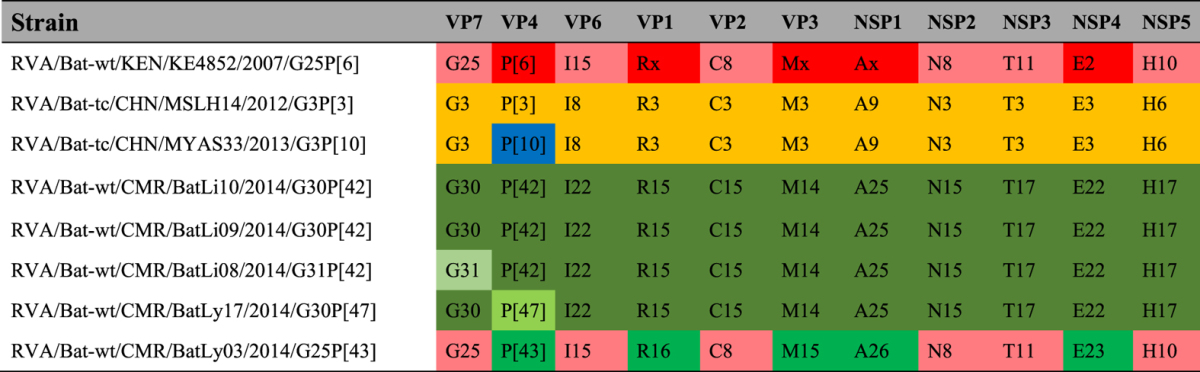
Genotype constellations of all known bat RVAs.

Pink indicates genotypes which are shared between Kenyan RVA strain KE4852 and BatLy03; red indicates (unknown) genotypes of KE4852; orange and blue indicate genotypes of Chinese bat RVA strains; and green shades represent all novel genotypes identified in Cameroonian bat RVAs.
