# Supplementary material for: Novel highly divergent reassortant bat rotaviruses in Cameroon, without evidence of zoonosis
Source: Sci Rep. 2016 Sep 26;6:34209. doi: 10.1038/srep34209 (PMC5035928; doi:10.1038/srep34209)
Supplement: Supplementary Information [file srep34209-s1.pdf]

Title

Novel highly divergent reassortant bat rotaviruses in Cameroon, without evidence of zoonosis.

Authors

Claude Kwe Yinda[1,2]; Mark Zeller[1]; Nádia Conceição-Neto[1,2]; Piet Maes[2]; Ward Deboutte[1]; Leen Beller[1]; Elisabeth Heylen[1]; Stephen Mbigha Ghogomu[3]; Marc Van Ranst[2]; Jelle Matthijnssens[1*]

[1]*KU Leuven - University of Leuven, Department of Microbiology and Immunology, Laboratory of Viral Metagenomics, Rega Institute for Medical Research, Leuven, Belgium*

[2]*KU Leuven - University of Leuven, Department of Microbiology and Immunology, Laboratory for Clinical Virology, Rega Institute for Medical Research, Leuven, Belgium*

[3]*University of Buea, Department of Biochemistry and Molecular Biology, Molecular and cell biology laboratory, Biotechnology Unit, Buea, Cameroon*

*Corresponding author.

Email addresses:

CKY: kweclaude.yinda@kuleuven.be

MZ: mark.zeller@kuleuven.be

NCN: nadia.neto@kuleuven.be

PM: piet.maes@kuleuven.be

WD: ward.deboutte@kuleuven.be

LB: leen.beller@kuleuven.be

EH: elisabeth.heylen@kuleuven.be

SMG: stephen.ghogomu@ubuea.cm

MVR: marc.vanranst@kuleuven.be

JM: jelle.matthijnssens@kuleuven.be

Supplementary data

Supplementary data S1: Nucleotide percentage identity of the novel bat RVAs and representative members of each genotype for each of the eleven segments.

VP7

G30_RVA/Bat-wt/CMR/BatLy17/2014/G30P[47]																																	
G25_RVA/Bat-wt/CMR/BatLy03/2014/G25P[43]	69																																
G31_RVA/Bat-wt/CMR/BatLi08/2014/G31P[42]	72	68																															
G30_RVA/Bat-wt/CMR/BatLi09/2014/G30P[42]	92	69	72																														
G30_RVA/Bat-wt/CMR/BatLi10/2014/G30P[42]	92	69	72	100																													
G3_RVA/Bat-wt/CHN/MYAS33/2013/G3P[10]	72	74	72	72	72																												
G3_RVA/Bat-tc/MSLH14/2012/G3P[3]	72	74	72	72	73	90																											
G1_RVA/Human-tc/USA/Wa/1974/G1P1A[8]	70	72	70	70	70	75	75																										
G2_RVA/Human-tc/USA/DS-1/1976/G2P1B[4]	70	69	67	71	71	74	73	74																									
G3_RVA/Human-tc/JPN/AU-1/1982/G3P3[9]	72	73	70	72	72	82	82	75	73																								
G4_RVA/Human-tc/GBR/ST3/1975/G4P2A[6]	71	72	69	71	71	75	74	76	70	75																							
G5_RVA/Pig-tc/USA/OSU/1977/G5P[7]	70	73	71	70	70	78	78	76	72	77	75																						
G6_Cow-tc/USA/NCDV/1971/G6P[1]	71	74	70	72	72	77	77	73	72	76	74	75																					
G7_RVA/Turkey-tc/IRL/Ty-3/1979/G7P[17]	63	62	62	64	64	64	65	63	62	63	64	64	62																				
G8_RVA/Human-tc/IND/69M/1980/G8P4[10]	70	73	69	70	70	77	76	71	73	75	71	75	74	62																			
G9_RVA/Human-tc/USA/WI61/1983/G9P1A[8]	72	75	72	72	72	79	79	75	73	78	76	78	75	63	75																		
G10_RVA/Human-tc/GBR/A64/1987/G10P[11][14]	70	72	71	70	70	76	78	74	71	74	72	73	75	64	74	76																	
G11_RVA/Pig-tc/MEX/YM/1983/G11P9[7]	70	74	70	70	70	79	79	73	75	78	74	82	75	62	76	78	73																
G12_RVA/Human-tc/PHL/L26/1987/G12P[4]	70	72	69	71	72	76	75	74	72	74	73	75	75	63	73	75	72	73															
G13_RVA/Horse-tc/GBR/L338/1991/G13P[18]	70	71	69	70	70	79	77	74	73	77	73	75	75	66	73	75	73	74	75														
G14_RVA/Horse-tc/USA/FI23/1981/G14P[12]	72	73	69	73	73	84	84	75	73	80	74	75	75	64	75	79	74	77	76	76													
G15_RVA/Cow-tc/IND/Hg18/1995/G15P[21]	70	72	69	70	70	73	74	73	71	72	72	74	74	64	72	74	72	74	71	72	73												
G16_RVA/Mouse-tc/USA/EW/XXXX/G16P[16]	71	71	67	71	71	78	77	71	70	75	72	73	75	62	75	73	71	74	73	73	75	71											
G17_RVA/Turkey-tc/IRL/Ty-1/1979/G17P[17]	65	65	63	63	63	65	66	64	64	64	64	65	62	69	63	66	64	65	64	65	66	64	62										
G18_RVA/Pigeon-tc/JPN/PO-13/1983/G18P[17]	61	64	63	61	61	63	64	63	61	65	62	64	62	70	62	65	63	62	64	63	65	62	63	77									
G19_RVA/Chicken-tc/GBR/Ch-1/197x/G19P[17]	63	63	64	63	63	65	65	63	62	66	63	63	61	67	63	65	64	64	64	64	64	62	61	72	73								
G20_RVA/Human-wt/ECU/Ecu534/2006/G20P[28]	69	72	69	70	70	76	76	72	70	75	71	75	74	62	74	73	73	75	74	73	74	70	73	65	64	63							
G21_RVA/Cow-wt/JPN/Azuk-1/2006/G21P[29]	72	72	68	71	72	73	73	74	71	72	70	73	72	62	71	72	72	72	72	73	73	72	71	64	63	63	71						
G22_RVA/Turkey-tc/DEU/03V0002E10/2003/G22P[35]	63	63	64	62	62	64	65	63	65	64	62	65	61	68	62	66	62	66	64	64	65	63	62	75	75	79	62	62					
G23_RVA/Pheasant-wt/HUN/Phea14246/2008/G23P[x]	63	63	63	64	64	66	66	65	65	65	65	64	63	73	64	65	65	63	64	65	66	64	61	70	69	70	64	63	70				
G24_RVA/Cow-tc/JPN/Dai-10/2007/G24P[33]	68	68	69	67	68	71	72	72	72	72	70	72	69	64	70	70	71	70	69	71	69	71	68	64	61	63	69	72	62	64			
G25_RVA/Bat-wt/KEN/KE4852/07/2007/G25P[6]	69	96	68	68	68	75	74	71	69	74	71	73	74	63	72	74	72	74	72	71	72	71	72	64	65	63	72	72	63	63	68		
G26_RVA/Pig-wt/JPN/TJ4-1/2010/G26P[7]	71	75	71	73	73	83	83	75	74	80	74	76	77	63	76	78	76	78	76	77	79	73	78	65	66	64	77	74	64	63	70	75	
G27_RVA/SugarGlider-tc/JPN/SG385/2012/G27P[36]	71	74	70	71	71	79	78	74	71	78	74	75	76	62	75	78	76	76	73	75	76	72	76	64	63	62	75	73	62	63	70	74	79

VP4

P[43]_RVA/Bat-wt/CMR/BatLy03/2014/G25P[43]
P[47]_RVA/Bat-wt/CMR/BatLy17/2014/G30P[47]	71
P[42]_RVA/Bat-wt/CMR/BatLi08/2014/G31P[42]	70	78
P[42]_RVA/Bat-wt/CMR/BatLi09/2014/G30P[42]	70	78	99
P[42]_RVA/Bat-wt/CMR/BatLi10/2014/G30P[42]	70	78	99	100
P[6]_RVA_Bat-wt/Kenya/KE4852/2007/G25P[6]	65	62	63	63	63
P[3]_RVA/Bat-tc/CHN/MSLH14/2012/G3P[3]	76	72	71	71	71	65
P[10]_RVA/Bat-wt/CHN/MYAS33/2013/G3P[10]	73	72	70	70	70	65	77
P[1]_RVA/Cow-wt//FRA/RF/xxxx/GxP[1]	74	70	71	71	71	65	76	
P[2]_RVA/LabStr/USA/SA11g4O-5N/XXXX/G3P[1]	75	72	72	72	72	65	79	76	
P[3]_RVA/Dog-tc/AUS/K9/1981/G3P[3]	73	71	71	71	71	64	82	76	76	78
P4_RVA/Human-tc/USA/DS-1/1976/G2P1B[4]	68	68	69	69	69	69	70	70	70	71	70
P5_RVA/Cow-tc/GBR/UK/1973/G6P7[5]	70	66	67	67	67	61	71	70	69	70	69	67
P[6]_RVA/Human-tc/GBR/ST3/1975/G4P2A[6]	70	68	68	68	68	93	72	71	70	71	71	73	67
P[7]_RVA/Pig-tc/USA/OSU/1977/G5P9[7]	72	72	70	70	70	63	76	74	75	74	76	69	68	69
P8_RVA/Human-tc/USA/Wa/1974/G1P1A[8]	69	69	68	68	68	71	70	70	69	70	70	87	67	75	68
P9_RVA/Human-tc/JPN/AU-1/1982/G3P3[9]	67	68	67	67	67	60	69	68	68	68	67	66	64	65	67	66
P[10]_RVA/Human-tc/IND/69M/1980/G8P4[10]	73	71	69	69	69	64	77	93	74	75	75	70	70	70	73	70	67
P[11]_RVA/Human-tc/IND/116E/1985/G9P[11]	61	60	59	59	59	50	61	61	60	59	60	60	59	59	61	59	59	61
P[12]_RVA/Horse-tc/GBR/H-2/1976/G3P[12]	73	71	71	71	71	64	75	78	74	75	74	71	70	70	73	72	69	78	61
P[13]_RVA/Pig-wt/xxx/A46/xxxx/GxP[13]	72	71	70	70	70	63	73	73	74	72	73	68	67	68	75	70	66	73	61	73
P[14]_RVA/Human-tc/GBR/A64/1987/G10P11[14]	66	68	66	66	66	58	68	67	66	66	67	64	66	65	68	65	77	67	58	68	67
P[15]_RVA/Sheep-wt/CHN/Lp14/xxxx/G10P[15]	75	71	70	70	70	65	77	74	74	75	74	70	71	70	73	70	67	73	61	73	72	67
P[16]_RVA/Mouse-tc/USA/EW/XXXX/G16P[16]P_16	68	66	66	67	67	59	70	70	68	70	71	66	67	66	69	66	63	69	57	69	69	63	69
P[17]_RVA/Pigeon-tc/JPN/PO-13/1983/G18P[17]	62	64	63	63	63	54	64	63	63	64	64	62	60	62	63	62	62	63	60	62	63	62	64	58
P[18]_RVA/Horse-tc/GBR/L338/1991/G13P[18]	74	72	72	72	72	67	76	74	77	76	75	70	71	71	75	71	67	74	60	74	73	68	74	69	63
P[19]_RVA/Human-tc/THA/Mc323/1989/G9P[19]	71	68	69	70	70	72	73	72	71	73	72	76	67	77	71	77	65	71	60	72	71	65	73	67	61	71
P[20]_RVA/Mouse-tc/XXX/EHP/1981/G16P[20]	69	67	66	66	66	63	71	72	71	70	71	66	68	67	70	67	63	72	56	70	69	62	70	72	61	70	68
P[21]_RVA/Cow-tc/IND/Hg18/1995/G15P[21]	72	70	69	69	69	63	74	73	73	73	74	70	69	69	71	70	65	73	60	72	71	66	74	66	63	73	72	69
P[22]_RVA/Rabbit-wt/ITA/160/01/GxP[22]	68	64	61	61	61	63	69	66	69	68	68	61	63	63	69	62	60	65	51	66	81	62	67	63	57	69	63	66	67
P[23]_RVA/Pig_wt/China/NMTL/2008/G9P[23]	74	72	71	71	71	67	77	75	74	75	76	71	71	72	75	71	67	74	61	74	72	68	75	70	63	74	73	70	72	68
P[24]_RVA/Rhesus-tc/USA/TUCH/2002/G3P[24]	74	71	71	71	71	65	77	76	74	76	76	70	70	70	74	71	66	75	61	75	73	66	77	70	63	75	73	71	74	67	76
P[25]_RVA/Human-wt/BGD/Dhaka6/2001/G11P[25]	67	68	66	66	66	60	69	67	67	67	69	66	65	66	67	66	73	67	58	69	67	72	67	63	61	68	67	64	66	62	68	67
P[26]_RVA/Pig-wt/ITA/134-04-15/2003/G5P[26]	72	70	70	70	70	63	75	74	74	74	74	69	69	68	75	69	66	74	59	73	80	68	72	68	64	74	70	70	72	76	73	73	68
P[27]_RVA/Pig-wt/THA/CMP034/2000/G2P[27]	71	68	69	69	69	64	70	71	71	71	70	68	67	68	69	69	65	71	58	71	70	65	71	66	62	71	69	67	70	64	71	72	63	70
P[28]_RVA/Human-wt/ECU/Ecu534/2006/G20P[28]	72	71	70	70	70	62	73	72	72	73	72	67	67	68	71	67	67	71	60	71	70	67	72	67	62	72	70	69	69	64	71	71	67	69	68
P[29]_RVA/Cow-wt/JPN/Azuk-1/2006/G21P[29]	64	64	64	65	65	55	66	65	66	65	66	63	63	63	65	64	63	65	60	65	65	63	64	61	60	65	65	62	65	57	66	65	64	65	64	65
P[30]_RVA/Chicken-tc/DEU/02V0002G3/2002/G19P[30]	63	64	63	64	64	55	64	63	63	65	63	62	60	63	63	63	62	63	60	64	63	61	64	58	77	64	61	60	63	56	64	63	61	63	62	62	61
P[31]_RVA/Chicken-tc/DEU/06V0661/2006/G19P[31]	63	63	63	63	63	57	64	62	63	65	64	62	59	63	63	62	61	61	59	63	63	60	63	59	75	64	62	60	63	55	65	63	61	64	63	62	61	76
P[32]_RVA/Pig-wt/IRL/61-07-ire/2007/G2P[32]	72	68	68	69	69	65	74	73	73	74	73	70	69	69	75	70	65	72	59	71	76	66	72	66	63	74	70	69	72	70	73	73	67	76	70	70	64	62	63
P[33]_RVA/Cow-tc/JPN/Dai-10/2007/G24P[33]	71	70	70	70	70	62	73	70	72	72	72	69	69	68	71	69	67	70	60	70	71	67	72	67	62	72	70	67	72	65	73	72	68	70	69	69	65	62	63	72
P[34]_RVA/Pig-wt/JPN/FGP51/2009/G4P[34]	70	68	68	68	68	64	72	71	72	70	71	69	67	69	69	69	65	71	59	72	69	64	71	66	62	71	71	68	70	63	73	71	65	69	79	68	64	61	63	69	70
P[35]_RVA/Turkey-tc/DEU/03V0002E10/2003/G22P[35]	63	63	63	63	63	55	64	64	63	64	63	63	60	63	63	63	61	63	60	63	63	61	64	60	78	64	62	60	63	57	63	62	60	64	62	62	60	77	75	63	64	62
P[36]_RVA/SugarGlider-tc/JPN/SG385/2012/G27P[36]	72	71	69	70	70	66	76	75	74	76	75	71	70	70	72	71	67	74	60	74	71	66	74	69	62	74	72	71	72	66	74	74	66	72	71	72	64	63	64	71	70	72	63
P[37]_RVA/Pheasant-tc/GER/10V0112H5/2010/G23P[37]	66	66	66	66	66	58	67	67	66	67	67	65	63	64	66	64	63	66	59	66	67	64	65	63	63	67	65	63	65	64	67	66	62	66	64	65	62	63	63	65	67	64	63	66
P[38]_RVA/Turkey-tc/IRL/Ty-1/1979/G17P[38]	63	64	63	64	63	56	64	63	64	65	64	62	60	63	63	63	62	63	62	63	64	60	63	60	77	64	63	61	64	58	64	64	62	64	62	62	62	75	73	64	63	61	76	63	63

VP6

I22_RVA/Bat-wt/CMR/BatLy17/2014/G30P[47]																										
I15_RVA/Bat-wt/CMR/BatLy03/2014/G25P[43]	75																									
I22_RVA/Bat-wt/CMR/BatLi08/2014/G31P[42]	99	75																								
I22_RVA/Bat-wt/CMR/BatLi09/2014/G30P[42]	99	75	99																							
I22_RVA/Bat-wt/CMR/BatLi10/2014/G30P[42]	99	75	99	100																						
I8_RVA/Bat-tc/CHN/MYAS33/2013/G3P[10]	76	80	76	76	75																					
I1_RVA/Human-tc/USA/Wa/1974/G1P1A[8]	75	78	75	75	75	80																				
I2_RVA/Human-tc/USA/DS-1/1976/G2P1B[4]	76	81	76	76	75	82	82																			
I3_RVA/Human-tc/JPN/AU-1/1982/G3P3[9]	76	81	76	76	75	81	78	81																		
I4_RVA/Pigeon-tc/JPN/PO-13/1983/G18P[17]	67	69	67	67	67	69	70	71	68																	
I5_RVA/Pig-tc/MEX/YM/1983/G11P9[7]	76	79	76	76	76	80	82	80	77	70																
I6_RVA/Horse-tc/GBR/L338/1991/G13P[18]	75	78	75	75	75	80	84	81	79	69	80															
I7_RVA/Mouse-tc/USA/EW/XXXX/G16P[16]	72	77	72	72	72	78	77	78	79	67	76	78														
I8_RVA/Human-wt/THA/CMH222/2001/G3P[3]	75	80	75	75	75	84	79	82	83	68	78	79	77													
I8_RVA/Bat-tc/MSLH14/2012/G3P[3]	75	80	75	75	75	88	79	82	82	69	79	79	78	86												
I9_RVA/Rhesus-tc/USA/TUCH/2002/G3P[24]	76	80	76	76	76	82	79	81	81	70	80	80	77	80	81											
I10_RVA/Sheep-wt/CHN/Lp14/xxxx/G10P[15]	76	81	76	76	76	82	81	85	81	70	79	80	78	81	81	83										
I11_RVA/Chicken-tc/DEU/02V0002G3/2002/G19P[30]	68	69	68	68	68	69	69	69	68	79	70	69	67	70	69	68	69									
I12_RVA/Human-wt/NPL/KTM368/2004/G11P[25]	75	77	75	75	75	79	83	80	79	69	85	81	76	79	79	81	79	70								
I13_RVA/Human-wt/ECU/Ecu534/2006/G20P[28]	77	74	77	77	77	75	74	75	75	69	76	75	72	75	74	75	74	68	74							
I14_RVA/Pig-wt/CAN/CE-M-06-0003/2005/G2P[27]	76	79	77	77	77	80	79	79	78	69	80	79	77	80	78	78	78	69	79	75						
I15_RVA/Bat-wt/KEN/KE4852/07/2007/G25P[6]	75	97	75	75	74	80	78	81	80	69	78	78	77	79	80	80	81	69	78	74	78					
I16_RVA/Human-tc/KEN/B10/1987/G3P[2]	76	81	76	76	76	85	80	82	81	70	79	80	78	82	83	82	82	70	79	75	78	81				
I17_RVA/Rabbit-tc/CHN/N5/1992/G3P[14]	75	79	75	75	75	83	79	82	82	69	78	78	76	82	83	83	82	69	79	75	79	79	82			
I18_RVA/Human-wt/BRA/QUI-35-F5/2010/G3P[9]	75	79	75	75	75	81	79	80	81	69	78	78	77	81	82	80	79	68	78	75	78	79	83	81		
I19_RVA/SugarGlider-wt/JPN/SG385/2012/G27P[36]	73	78	73	73	73	79	78	80	78	68	78	78	77	78	79	81	78	68	77	75	76	77	80	79	78	
I20_RVA/Rat-wt/GER/KS-11-573/2011/G3P[3]	72	78	72	72	72	80	78	79	80	68	78	78	78	80	79	78	79	66	77	73	77	79	81	79	78	77

VP1

R15_RVA/Bat-wt/CMR/BatLy17/2014/G30P44																			
R16_RVA/Bat-wt/CMR/BatLy03/2014/G25P[43]	74																		
R15_RVA/Bat-wt/CMR/BatLi08/2014/G31P[42]	99	75																	
R15_RVA/Bat-wt/CMR/BatLi09/2014/G30P[42]	99	75	99																
R15_RVA/Bat-wt/CMR/BatLi10/2014/G30P[42]	99	74	99	100															
R3_RVA/Bat-tc/CHN/MYAS33/2013/G3P[10]	74	78	75	75	74														
R3_RVA/Bat-tc/MSLH14/2012/G3P[3]	73	78	73	73	73	84													
R1_RVA/Human-tc/USA/Wa/1974/G1P1A[8]	74	78	74	74	74	80	80												
R2_RVA/Human-tc/USA/DS-1/1976/G2P1B[4]	74	79	74	74	74	81	80	79											
R3_RVA/Human-tc/JPN/AU-1/1982/G3P3[9]	74	78	74	74	74	88	85	80	81										
R4_RVA/Pigeon-tc/JPN/PO-13/1983/G18P[17]	72	72	72	72	72	72	71	71	73	71									
R5_RVA/Guanaco-wt/ARG/Chubut/1999/G8P[14]	74	77	74	74	74	80	80	79	82	80	72								
R6_RVA/Chicken-tc/DEU/02V0002G3/2002/G19P[30]	71	71	71	71	71	72	71	71	72	71	79	72							
R7_RVA/Mouse-tc/USA/ETD_822/XXXX/G16P[16]	71	74	71	71	71	75	74	74	76	74	69	74	68						
R8_RVA/Human-tc/KEN/B10/1987/G3P[2]	74	78	74	74	74	82	82	81	81	82	72	80	72	74					
R9_RVA/Horse-tc/GBR/L338/1991/G13P[18]	75	77	75	75	74	80	79	78	80	79	72	81	72	73	79				
R10_RVA/SugarGlider-wt/JPN/SG385/2012/G27P[36]	74	76	74	74	73	79	79	78	79	80	71	78	71	74	79	78			
R11_RVA/Rat-wt/GER/KS-11-573/2011/G3P[3]	72	76	73	72	72	80	81	78	78	81	69	78	70	74	80	78	78		
R12_RVA/Human-wt/ITA/ME848/12/2012/G12P[9]	75	78	75	75	75	81	81	81	79	81	72	80	72	74	81	80	79	79	
RR13_RVA/Human-xx/USA/2014735512/xxxx/G20P[28]	76	75	76	76	75	75	74	76	75	75	72	75	72	72	75	75	75	74	76

VP2

C8_RVA/Bat-wt/CMR/BatLy03/2014/G25P[43]																			
C15_RVA/Bat-wt/CMR/BatLy17/2014/G30P44	77																		
C15_RVA/Bat-wt/CMR/BatLi08/2014/G31P[42]	77	99																	
C15_RVA/Bat-wt/CMR/BatLi09/2014/G30P[42]	77	99	100																
C15_RVA/Bat-wt/CMR/BatLi10/2014/G30P[42]	77	99	100	100															
C3_RVA/Bat-tc/CHN/MYAS33/2013/G3P[10]	79	78	78	78	78														
C3_RVA/Bat-wt/CHN/MSLH14/2013/G3P[3]	79	78	78	78	78	88													
C1_RVA/Human-tc/USA/Wa/1974/G1P1A[8]	80	78	78	78	78	80	79												
C2_RVA/Human-tc/USA/DS-1/1976/G2P1B[4]	80	76	76	76	76	80	81	80											
C3_RVA/Human-tc/JPN/AU-1/1982/G3P3[9]	79	77	78	78	78	90	88	79	81										
C4_RVA/Pigeon-tc/JPN/PO-13/1983/G18P[17]	68	70	70	70	70	70	70	69	69	69									
C5_RVA/LabStr/USA/SA11g4O-5N/XXXX/G3P[1]	80	78	78	78	78	82	82	81	81	83	69								
C6_RVA/Chicken-tc/DEU/02V0002G3/2002/G19P[30]	71	73	73	73	73	71	72	73	72	72	76	72							
C7_RVA/Rat-tc/ETD_822/2007/GxP[x]	74	72	73	73	73	75	75	75	74	76	68	74	68						
C8_RVA/Bat-wt/KEN/KE4852/07/2007/G25P[6]	98	76	77	76	76	78	78	80	80	78	68	80	71	74					
C9_RVA/Horse-tc/GBR/L338/1991/G13P[18]	79	78	79	79	79	81	81	80	81	81	71	80	72	75	79				
C10-RVA/SugarGlider-tc/JPN/SG385/2012/G27P[36]	78	78	78	78	78	81	80	80	81	81	68	80	71	75	79	79			
C11-RVA/Rat-wt/GER/KS-11-573/2011/G3P[3]	76	76	76	76	76	80	79	77	77	80	69	78	69	73	76	79	77		
C12-RVA/Hu/ITA/ME848-12/2012/G12P[8]	79	78	78	78	78	81	81	81	81	82	69	82	72	75	79	81	81	79	
C13-RVA/Human-xx/USA/2014735512/xxxx/G20P[28]	76	77	77	77	77	78	77	77	77	78	69	79	72	73	76	78	77	75	77

VP3

M14_RVA/Bat-wt/CMR/BatLy17/2014/G30P[47]																		
M15_RVA/Bat-wt/CMR/BatLy03/2014/G25P[43]	68																	
M14_RVA/Bat-wt/CMR/BatLi08/2014/G31P[42]	90	68																
M14_RVA/Bat-wt/CMR/BatLi09/2014/G30P[42]	90	68	100															
M14_RVA/Bat-wt/CMR/BatLi10/2014/G30P[42]	90	68	100	100														
M3_RVA/Bat-tc/CHN/MYAS33/2013/G3P[10]	67	71	67	67	67													
M3_RVA/Bat-tc/CHN/MSLH14/2012/G3P[3]	67	71	66	66	66	95												
M9-RVA/SugarGlider-tc/JPN/SG385/2012/G27P[36]	67	69	67	67	67	72	72											
M8-RVA/Mouse-tc/USA/ETD_822/XXXX/G16P[16]	63	66	63	63	63	67	67	67										
M7_RVA/Chicken-tc/DEU/02V0002G3/2002/G19P[30]	62	65	62	62	62	62	61	61	59									
M6_RVA/Horse-tc/GBR/L338/1991/G13P[18]	68	72	68	68	68	76	76	72	68	62								
M5_RVA/Simian-tc/ZAF/SA11-H96/1958/G3P5B2	69	71	69	69	69	78	79	72	69	63	77							
M4_RVA/Pigeon-tc/JPN/PO-13/1983/G18P[17]	61	63	61	61	61	60	61	59	58	73	62	62						
M3_RVA/Human-tc/JPN/AU-1/1982/G3P3[9]	67	71	67	67	67	87	87	72	68	61	76	80	61					
M2_RVA/Human-tc/USA/DS-1/1976/G2P1B[4]	69	71	68	68	68	76	76	71	68	62	78	78	62	75				
M12-RVA/Human-xx/USA/2014735512/xxxx/G20P[28]	69	68	67	67	67	69	69	69	64	64	71	70	62	69	71			
M11-RVA/Human-xx/ITA/ME848-12/2012/G12P[8]	68	70	67	67	67	77	77	71	67	63	77	78	62	77	76	70		
M10-RVA/Rat-wt/GER/KS-11-573/2011/G3P[3]	66	69	66	66	66	76	76	69	68	61	74	77	59	76	74	68	75	
M1_RVA/Human-tc/USA/Wa/1974/G1P1A[8]	68	71	68	68	68	77	76	72	67	63	79	78	61	77	77	70	77	74

NSP1

A25_RVA/Bat-wt/CMR/BatLy17/2014/G30P[47]																																
A26_RVA/Bat-wt/CMR/BatLy03/2014/G25P[43]	67																															
A25_RVA/Bat-wt/CMR/BatLi10/2014/G30P[42]	99	67																														
A25_RVA/Bat-wt/CMR/BatLi08/2014/G31P[42]	99	67	98																													
A25_RVA/Bat-wt/CMR/BatLi09/2014/G30P[42]	99	67	100	98																												
A9_RVA/Bat-tc/CHN/MSLH14/2012/G3P[3]	42	42	42	42	42																											
A9_RVA/Bat-tc/CHN/MYAS33/2013/G3P[10]	42	42	42	42	42	94																										
A1_RVA/Human-tc/USA/Wa/1974/G1P1A[8]	43	43	43	43	43	51	50																									
A1_RVA/Human-tc/USA/Wa/1974/G1P1A[8]	43	43	43	43	43	51	50	100																								
A2_RVA/Human-tc/USA/DS-1/1976/G2P1B[4]	43	43	43	42	43	51	50	75	75																							
A3_RVA/Human-tc/JPN/AU-1/1982/G3P3[9]	41	40	41	41	41	49	49	64	64	64																						
A4_RVA/Pigeon-tc/JPN/PO-13/1983/G18P[17]	34	36	34	34	34	34	33	37	37	37	35																					
A5_RVA/Simian-tc/ZAF/SA11-H96/1958/G3P5B2	43	42	43	43	43	61	62	52	52	52	50	37																				
A6_RVA/Horse-tc/GBR/L338/1991/G13P[18]	43	42	43	43	43	71	71	51	51	50	51	36	62																			
A7_RVA/Mouse-tc/USA/EW/XXXX/G16P[16]	39	40	39	39	39	56	56	50	50	48	48	34	54	55																		
A8_RVA/Pig-tc/MEX/YM/1983/G11P9[7]	43	42	43	42	43	49	50	77	77	73	66	36	50	50	49																	
A9_RVA/Human-wt/BEL/B4106/2000/G3P[14]	41	43	41	41	41	83	83	51	51	51	49	35	62	72	56	48																
A10_RVA/Horse-tc/xxx/FI14/xxxx/GxP[x]	43	44	43	43	43	72	72	51	51	51	50	36	62	77	55	50	72															
A11_RVA/Human-wt/HUN/Hun5/1997/G6P[14]	43	42	43	43	43	48	48	65	65	65	72	37	50	51	50	65	49	50														
A12_RVA/Human-tc/THA/T152/1998/G12P[9]	41	41	41	41	41	50	51	61	61	60	59	36	55	51	48	59	52	52	60													
A13_RVA/Cow-tc/USA/B223/XXXX/G10P[11]	44	42	44	44	44	49	49	66	66	65	72	36	52	51	49	65	50	52	71	61												
A14_RVA/Cow-tc/THA/A5-13/XXXX/G8P[1]	45	43	45	45	45	49	49	67	67	68	72	36	52	52	48	66	50	52	71	62	79											
A15_RVA/Human-tc/ITA/PA260-97/1997/G3P[3]	41	41	41	40	41	50	51	59	59	59	59	36	52	51	49	58	52	51	60	65	61	61										
A16_RVA/Chicken-tc/DEU/02V0002G3/2002/G19P[30]	35	35	35	35	35	37	36	37	37	37	35	63	39	36	34	36	36	36	37	35	35	35	37									
A17_RVA/alpaca-wt/PER/356/2010/G3P[14]	43	43	43	43	43	74	73	53	53	53	51	36	63	71	53	50	73	72	51	53	51	53	52	37								
A18_RVA/Camel-wt/SDN/MRC-DPRU447/2009/G8P[1]	43	42	43	43	43	51	50	65	65	64	68	36	51	53	50	63	51	52	68	60	70	74	59	36	53							
A19_RVA/Human-wt/BRA/QUI-35-F5/2010/G3P[9]	42	42	42	42	42	50	50	59	59	59	61	37	51	53	49	59	51	53	60	65	61	61	66	37	54	60						
A20_RVA/SugarGlider-tc/JPN/SG385/2012/G27P[36]	41	40	41	41	41	53	53	56	56	58	56	37	52	52	48	56	52	53	56	59	57	57	62	36	53	56	61					
A21_RVA/VelvetScoter-tc/JPN/RK1/1989/G18P[17]	33	33	32	32	32	32	32	36	36	37	35	52	34	34	32	35	33	33	37	34	37	37	34	51	34	37	34	34				
A22_RVA/Rat-wt/GER/KS-11-573/2011/G3P[3]	41	43	41	41	41	71	70	49	49	50	49	35	61	68	56	47	70	68	50	51	50	49	49	35	68	50	50	51	33			
A23_RVA/Human-xx/USA/2014735512/xxxx/G20P[28]	42	43	41	42	41	50	50	59	59	58	60	38	51	52	49	57	51	51	60	64	59	60	66	36	53	59	76	62	34	50		
A24_RVA/CommonGull-wt/JPN/Ho374/2013/G28P3[9]	34	33	34	34	34	34	34	35	35	36	35	52	34	34	33	35	35	34	36	35	35	36	36	52	35	37	36	36	66	32	35	

NSP2

N15_RVA/Bat-wt/CMR/BatLy17/2014/G30P[47]																			
N8_RVA/Bat-wt/CMR/BatLy03/2014/G25P[43]	71																		
N15_RVA/Bat-wt/CMR/BatLi08/2014/G31P[42]	96	71																	
N15_RVA/Bat-wt/CMR/BatLi09/2014/G30P[42]	97	72	99																
N15_RVA/Bat-wt/CMR/BatLi10/2014/G30P[42]	97	72	99	100															
N3_RVA/Bat-tc/MSLH14/2012/G3P[3]	71	75	70	71	71														
N3_RVA/Bat-tc/CHN/MYAS33/2013/G3P[10]	71	75	69	70	70	95													
N8_RVA/Bat-wt/KEN/KE4852/07/2007/G25P[6]	71	97	70	71	71	74	75												
N1_RVA/Human-tc/USA/Wa/1974/G1P1A[8]	74	78	73	74	74	81	81	77											
N2_RVA/Human-tc/USA/DS-1/1976/G2P1B[4]	71	75	70	71	71	80	80	74	82										
N3_RVA/Human-tc/JPN/AU-1/1982/G3P3[9]	71	74	69	71	71	87	87	74	79	80									
N4_RVA/Pigeon-tc/JPN/PO-13/1983/G18P[17]	63	62	62	63	63	61	61	62	61	61	61								
N5_RVA/Simian-tc/ZAF/SA11-H96/1958/G3P5B2	72	76	72	73	73	82	82	75	81	78	82	60							
N6_RVA/Chicken-tc/DEU/02V0002G3/2002/G19P[30]	64	61	63	64	64	61	61	62	63	59	60	77	61						
N7_RVA/Mouse-tc/USA/ETD_822/XXXX/G16P[16]	68	73	67	68	68	74	74	74	72	72	73	59	74	58					
N9_RVA/Horse-tc/GBR/L338/1991/G13P[18]	72	77	70	72	72	80	80	76	80	81	78	62	78	61	72				
N10_RVA/pheasant-tc/GER/10V0112H5/2010/G23P[37]	65	62	64	65	65	61	62	62	63	61	62	79	62	85	59	62			
N11_RVA/SugarGlider-tc/JPN/SG385/2012/G27P[36]	70	76	69	70	70	77	76	76	78	76	76	61	78	60	72	77	60		
N12_RVA/Hu/ITA/ME848-12/2012/G12P[8]	71	77	70	71	71	80	81	77	81	79	80	61	78	61	74	77	63	77	
N13_RVA/Human-xx/USA/2014735512/xxxx/G20P[28]	71	68	70	72	72	67	68	67	69	69	67	62	67	63	67	69	63	68	69

NSP3

T17_RVA/Bat-wt/CMR/BatLy17/2014/G30P[47]																					
T11_RVA/Bat-wt/CMR/BatLy03/2014/G25P[43]	69																				
T17_RVA/Bat-wt/CMR/BatLi08/2014/G31P[42]	98	69																			
T17_RVA/Bat-wt/CMR/BatLi09/2014/G30P[42]	99	69	99																		
T17_RVA/Bat-wt/CMR/BatLi10/2014/G30P[42]	99	69	99	100																	
T3_RVA/Bat-wt/CHN/MYAS33/2013/G3P[10]	71	71	72	72	71																
T3_RVA/Bat-tc/MSLH14/2012/G3P[3]	70	70	70	70	70	87															
T1_RVA/Human-tc/USA/Wa/1974/G1P1A[8]	70	72	71	70	70	77	77														
T2_RVA/Human-tc/USA/DS-1/1976/G2P1B[4]	70	70	71	70	70	83	83	77													
T3_RVA/Human-tc/JPN/AU-1/1982/G3P3[9]	70	71	71	71	71	85	85	76	82												
T4_RVA/Pigeon-tc/JPN/PO-13/1983/G18P[17]	54	54	55	54	54	55	56	55	55	56											
T5_RVA/Simian-tc/ZAF/SA11-H96/1958/G3P5B2	69	70	70	69	69	77	77	75	76	78	55										
T6_RVA/Cow-tc/USA/WC3/1981/G6P[5]	69	72	70	70	69	79	77	82	78	78	55	76									
T7_RVA/Cow-tc/GBR/UK/1973/G6P7[5]	69	74	70	70	69	78	77	84	78	77	56	73	83								
T8_RVA/Chicken-tc/DEU/02V0002G3/2002/G19P[30]	53	54	53	53	53	55	55	55	56	56	67	55	55	56							
T9_RVA/Cow-wt/JPN/Azuk-1/2006/G21P[29]	71	72	71	71	71	77	77	76	76	77	53	76	77	77	54						
T10_RVA/Mouse-tc/USA/ETD_822/XXXX/G16P[16]	63	65	64	63	63	70	69	69	68	69	53	67	68	70	53	69					
T11_RVA/Bat-wt/KEN/KE4852/07/2007/G25P[6]	68	96	69	68	68	71	70	72	70	71	55	70	72	73	54	72	65				
T12_RVA/Horse-tc/GBR/L338/1991/G13P[18]	70	69	70	69	69	73	76	71	72	74	55	72	73	73	55	73	67	69			
T13_RVA/SugarGlider-wt/JPN/SG385/2012/G27P[36]	71	71	72	71	71	78	79	76	78	80	56	74	75	76	56	75	67	72	73		
T14_RVA/Rat-wt/GER/KS-11-573/2011/G3P[3]	70	71	71	70	70	79	79	77	80	81	56	75	75	77	54	75	69	71	72	78	
T15_RVA/Human-xx/USA/2014735512/xxxx/G20P[28]	70	69	69	70	70	73	71	72	72	73	57	69	73	72	55	71	67	69	70	71	71

NSP4

E22_RVA/Bat-wt/CMR/BatLy17/2014/G30P[47]																											
E23_RVA/Bat-wt/CMR/BatLy03/2014/G25P[47]	62																										
E22_RVA/Bat-wt/CMR/BatLi08/2014/G31P[42]	100	63																									
E22_RVA/Bat-wt/CMR/BatLi09/2014/G30P[42]	100	62	100																								
E22_RVA/Bat-wt/CMR/BatLi10/2014/G30P[42]	100	62	100	100																							
E2_RVA/Bat-wt/KEN/KE4852/2007/G25P[6]	65	67	65	65	65																						
E3_RVA/Bat-tc/CHN/MYAS33/2013/G3P[10]	66	69	67	66	66	78																					
E3_RVA/Bat-tc/MSLH14/2012/G3P[3]	66	69	67	66	66	79	91																				
E1_RVA/Human-tc/USA/Wa/1974/G1P1A[8]	65	67	66	65	65	80	83	82																			
E3_RVA/Human-tc/JPN/AU-1/1982/G3P3[9]	65	68	66	65	65	79	89	91	81																		
E4_RVA/Pigeon-tc/JPN/PO-13/1983/G18P[17]	49	50	51	49	49	52	53	52	51	52																	
E5_RVA/Human-wt/BEL/B4106/2000/G3P[14]	63	68	65	63	63	81	79	78	79	77	54																
E6_RVA/Human-wt/BGD/N26/2002/G12P[6]	66	67	67	66	66	79	83	83	81	82	51	79															
E7_RVA/Mouse-tc/USA/EW/XXXX/G16P[16]	59	58	61	59	59	66	66	66	64	67	47	66	65														
E8_RVA/Cow-lab/GBR/PP-1/1976/G3P[7]	62	62	64	62	62	73	72	72	76	72	47	71	72	62													
E9_RVA/Pig-wt/THA/CMP034/2000/G2P[27]	64	68	66	64	64	75	77	76	82	76	51	77	77	64	72												
E10_RVA/Panda-xx/CHN/CH-1/2008/G1P[7]	54	52	55	54	54	51	53	53	52	51	75	53	53	51	49	62											
E11_RVA/Turkey-tc/IRL/Ty-1/1979/G17P[17]	49	49	50	49	49	49	52	50	48	51	81	49	50	47	46	62	76										
E12_RVA/Guanaco-wt/ARG/Chubut/1999/G8P[14]	65	68	66	65	65	83	80	80	80	78	51	82	80	65	75	62	52	50									
E13-RVA/Human-tc/KEN/B10/1987/G3P[2]	68	70	69	68	68	77	82	80	76	80	51	75	78	66	69	62	54	51	77								
E14-RVA/Horse-tc/GBR/L338/1991/G13P[18]	67	66	68	67	67	75	74	74	77	75	52	74	77	63	68	62	54	52	77	72							
E15-RVA/camel-xx/KUW/s21/2010/G10P[15]	61	60	62	61	61	67	66	66	66	63	49	68	67	55	59	62	47	45	68	64	66						
E16_RVA/Alpaca-wt/PER/AlP[11]A/2010/G8P[x]	63	66	64	63	63	72	80	79	74	78	48	72	78	62	64	62	51	50	73	75	72	66					
E16_RVA/Vicuna-wt/ARG/C75/2010/G8P[14]	62	67	63	62	62	72	81	81	75	80	48	72	78	63	65	62	51	49	73	74	70	65	89				
E17_RVA/SugarGlider-wt/JPN/SG385/2012/G27P[36]	64	66	65	64	64	73	81	79	75	79	53	75	77	64	70	62	52	49	76	77	72	63	73	73			
E18_RVA/Rat-wt/GER/KS-11-573/2011/G3P[3]	61	67	62	61	61	70	76	74	73	74	50	71	75	63	65	62	51	48	71	73	68	61	70	70	70		
E19_RVA/Fox-wt/ITA/288356/2011/G18P[17]	52	52	53	52	52	50	50	51	48	51	77	51	50	47	47	62	74	76	50	52	53	47	49	48	51	50	
E20_RVA/Human-xx/USA/2014735512/xxxx/G20P[28]	66	61	67	66	66	65	65	66	68	66	51	66	68	59	61	62	53	52	64	65	64	58	65	63	65	62	50

NSP5

H17_RVA/Bat-wt/CMR/BatLy17/2014/G30P[47]																				
H10_RVA/Bat-wt/CMR/BatLy03/2014/G25P[43]	73																			
H17_RVA/Bat-wt/CMR/BatLi08/2014/G31P[42]	99	74																		
H17_RVA/Bat-wt/CMR/BatLi09/2014/G30P[42]	99	74	100																	
H17_RVA/Bat-wt/CMR/BatLi10/2014/G30P[42]	99	73	100	100																
H6_RVA/Bat-wt/CHN/MYAS33/2013/G3P[10]	74	81	75	75	74															
H6_RVA/Bat-tc/MSLH14/2012/G3P[3]	74	81	74	74	74	97														
H1RVA/Human-tc/USA/Wa/1974/G1P1A[8]	74	82	75	75	75	88	87													
H2_RVA/Human-tc/USA/DS-1/1976/G2P1B[4]	73	79	74	74	73	84	84	83												
H3_RVA/Human-tc/JPN/AU-1/1982/G3P3[9]	72	81	73	74	73	88	87	87	84											
H4_RVA/Pigeon-tc/JPN/PO-13/1983/G18P[17]	58	61	59	58	59	63	62	63	61	62										
H5_RVA/LabStr/USA/SA11g4O-5N/XXXX/G3P[1]	74	84	75	75	74	90	90	88	85	89	62									
H6_RVA/Human-tc/THA/T152/1998/G12P[9]	73	81	74	74	74	93	93	87	84	88	63	90								
H7_RVA/Horse-wt/ARG/E30/1993/G3P[12]	73	82	74	74	74	83	83	82	80	84	59	84	82							
H9-RVA/Mouse-tc/USA/ETD_822/2007/G16P[16]	69	76	69	69	68	78	78	76	77	77	58	79	78	76						
H10-RVA/Bat-wt/KEN/KE4852/07/2007/G25P[6]	72	96	72	72	72	81	81	82	79	82	60	84	81	81	76					
H11-RVA/Horse-tc/GBR/L338/1991/G13P[18]	72	79	73	73	73	84	83	83	81	83	63	83	84	82	75	79				
H12_RVA/SugarGlider-wt/JPN/SG385/2012/G27P[36]	72	79	73	73	72	85	85	82	81	85	62	85	84	81	75	79	82			
H13_RVA/Rat-wt/GER/KS-11-573/2011/G3P[3]	71	80	72	72	71	85	84	84	82	83	59	86	84	82	77	80	79	79		
H14-RVA/Turkey-tc/IRL/Ty-3/1979/G7P[17]	60	61	60	60	60	63	62	62	60	61	84	63	63	60	58	62	62	63	59	
H15_R13_RVA/Human-xx/USA/2014735512/xxxx/G20P[	72	71	72	73	72	76	76	75	73	74	59	76	75	72	69	71	73	75	73	61

Supplementary data S2: Nucleotide percentage similarity between primers and corresponding sequences of Cameroonian bat RVA strains. -: 5' or 3'-end amplification failed.

**Name of primer**	**BatLy03 G25P[43]**	**BatLi08 G31P[42]**	**BatLi09 G30P[42]**	**BatLy17 G30P[47]**	**BatLi10 G30P[42]**	**Reference (PMCID)**
**Outer capsid glycoprotein VP7 forward primers**						
Beg9	96.4	57.1	-	85.7	-	2155916
sBeg9	100	57.1	-	90.5	-	2155916
9Con1-L	100	100	-	100	-	7929782
**Outer capsid glycoprotein VP7 reverse primer**						
EndA	78.2	91.3	95.7	-	-	10364621
VP7-Rdeg	95.0	95.0	100	-	-	11264359
End9	85.1	66.7	59.3	-	-	2155916
RVG9	89.5	63.2	63.2	-	-	2155916
**Outer capsid protein VP4 forward primers**						
VP4-1-17F	88.8	88.8	77.8	94.4	-	22189107
Con3	72.7	68.2	72.7	63.6	72.7	1320625
**Outer capsid protein VP4 reverse primer**						
Con2	85.7	81.0	81.0	76.2	81.0	1320625

Supplementary data S3: Phylogenetic trees of nucleotide sequences of the RVA VP6 sequence. HRVA: Human RVA VP6 sequence from patients exposed to bats; open triangle: Cameroonian bat RVA strains; Other bat RVA: KE4582, MSLH14 and MYAS33. Bootstrap values (1000 replicates) above 70% are shown. Scale represent number of nucleotide substations per site.

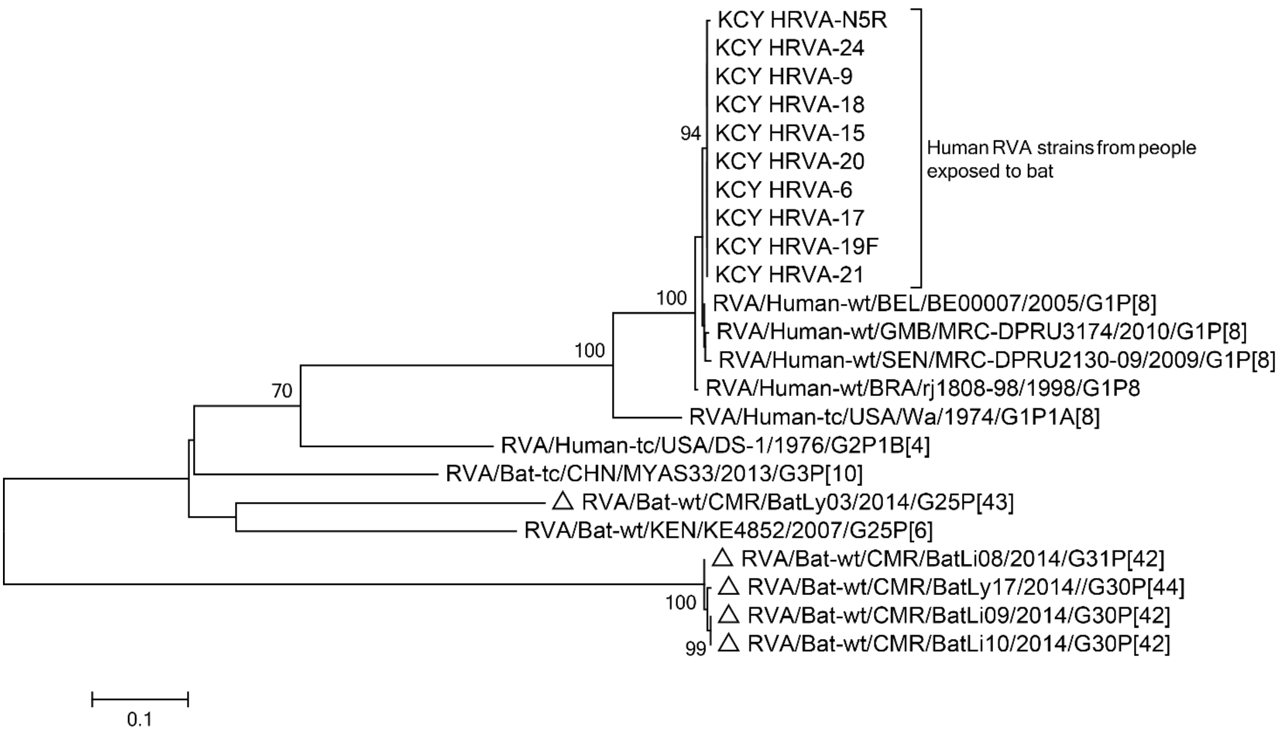

Supplementary data S4: List of primers used for the completion of genome and screening

Description	Primer name	Primer sequence (5'→3')
Screening primers for human and bat RVAs	RVA-VP6_40F	GCK AGR GAC AAR ATT GTY GAA GG
	RVA-VP6_1083R	GWC CAA TTC ATR CCT GGT GGA
Primers for completing segments of RVA/Bat-wt/CMR/BatLy03/2014/G25P[43]	RVA59_VP1_301-322R	TTA GCT AAT TTA CCC TCA ACG G
	RVA59_VP1_328-349F	AAG CTA ACG TCA GAA TTA TTC GC
	RVA59_VP1_737-758R	GCT ACT AAG ATT GAC ATT GGT G
	RVA59_VP1_2991-3013F	GTC AAT AAA CTA TGG CTG TTA CC
	RVA59_VP1_3048-3072F	ATT AAT TCG TAT ACC CTT CAA AGG T
	RVA59_VP3_76-100F	CAA TCT TAT GCT GAT ACT CAA ACG
	RVA59_VP3_787-809R	TGT CAA ATT GTG AAA TAC GTC GC
	RVA59_VP3_1795-1818F	CGT TAT CTG GTT ACA TAT TTA GAG
	RVA59_VP4_2063-2084F	GAA CAG ATG GTA GAT TTT TCG C
	RVA59_VP4_171-195R	TAC AGT TGT TGA ATC GTT AAT TTC C
	RVA59_NSP1_1433-1453F	AAA TCA CTC TTA TTC CGG TGG
	RVA59_NSP5_389-411F	ATC AAT TAC TAC GGA TCA TGC TG
Primers for completing segments of RVA/Bat-wt/CMR/BatLi10/2014/G30P[42]	RVA46_VP4_118-137_F	TTA CGC ACC AGT GAA TTG GG
	RVA46_VP4_839-860_R	ACC ACT TAT ATC CTA AAC CAC C
	RVA46_VP7_172-191_F	TCA ATG GAC GTT GTG TTG GC
	RVA46_VP7_803-823_R	GAT TTG TTG TTG GAT CTG ACG
Primers for the amplification of 5'and 3' ends of VP7 and VP4 segments	RVA63_VP7-5'-227_R	TTA CTAGAC TCA TGT CCA TCG
	RVA63_VP7-3'-839_F	AGT ATT ACA AGT TGG AGG TGC
	RVA894_VP7-5'-202_R	TGT AAC CGG TAT GTT CAA TCC
	RVA894_VP7-3'-788_F	GTA AGA AAA TCG GAC CTA GAG
	RVA59_VP7-5'-213_R	TCC ATT GAT CCA GTA ATT GGC
	RVA59_VP7-3'-843_F	TAT AAC AGC AGA TCC AAC GAC
	RVA63_VP4-5'-230_R	ATA CCC AAT TAT CGA CTG CTG
	RVA63_VP4-3'-2143_F	CTT GTA ACT GAT TCA CCA GTC
	RVA59_VP4-5'-214_R	AAA TTC TGT TGG CTG ATA GGG
	RVA59_VP4-3'-2100_F	AGA TTT TTC GCA TAT AAA GTG AGC
	RVA894_VP4-5'-158_R	TAC TGT TGA GTC ATT TAC TTC CC
	RVA894_VP4-3'-2072_F	GAA CAG ATG GTA GAT TTT TCG C
